# Rethinking the World Health Organization’s leadership of global health governance and the global health surveillance systems

**DOI:** 10.1177/17579759231220529

**Published:** 2024-01-29

**Authors:** Mohammed Alkhaldi, Hamza Meghari, Marina AlBada

**Affiliations:** 1 McGill University, Faculty of Medicine, and Health Sciences, School of Physical and Occupational Therapy, Montreal, Canada; 2 McGill University, McGill University Health Centre (MUHC), Montreal, Canada; 3 Canadian Institutes of Health Research (CIHR), Health Systems Impact Fellowship, Ottawa, Canada; 4 Canadian University Dubai, Department of Public Health, Faculty of Communication, Arts and Sciences, UAE; 5 University of Basel, Swiss Tropical and Public Health Institute (Swiss TPH), Switzerland; 6 The University of Oxford, Nuffield Department of Medicine, United Kingdom; 7 University College London UCL, Institute of Child Health, Infection, Immunity, and Inflammation Department, United Kingdom; 8 The University of Edinburgh, Global Health Policy (MSc), School of Social and Political Science, United Kingdom

**Keywords:** global health governance, public health surveillance systems, WHO’s mandate

## Abstract

Global health governance is a strategic priority for the World Health Organization (WHO), and the public health surveillance system (PHSS) is a fundamental element of the global health governance structure to timely identify emerging diseases and guide global public health decisions and actions. This analysis explores the overall landscape of global health governance, with a specific focus on the PHSS to understand whether the existing governance landscape facilitates or undermines the WHO’s ability to formulate and implement global health policies and initiatives. To achieve this, the existing evidence was reviewed, and synthesized with the experts’ perspectives. It is reported that fragmentation is the main drawback of the global health governance landscape, necessitating reorganization and restructuring. The disintegration of PHSS at the global, regional and local levels is associated with a lack of leadership, misalignment with global health priorities, imbalance in coverage of surveillance systems, inadequate innovative technology and digitalization, and fragmented data and information systems. The fragmentation and disintegration of global health governance undermine the effectiveness of the WHO’s global health strategic directions and programmes and hinder its ability to govern and guide the global, regional and national public health emergency response. Strategic rethinking of the WHO’s governance is essential because strong governance and leadership lead to a robust, aligned and effective PHSS.

## Introduction

The World Health Organization (WHO) is at the forefront of the global response to health threats and pandemics, with a mandate to direct and coordinate global actions for achieving the highest attainable standard of health. Strengthening the global governance for health emergency preparedness, response and resilience (HEPR) requires effective leadership, inclusivity and accountability. The WHO is governing these actions by adhering to international legal instruments, such as the International Health Regulations (2005) (1), and through initiatives like the Intergovernmental Negotiating Body, which focuses on strengthening pandemic prevention, preparedness and response (2,3). Furthermore, the establishment of the Standing Committee on Health Emergency Prevention, Preparedness, and Response reinforces the WHO’s ability to address health emergencies. Proposals for a global health threats council and a high-level meeting at the United Nations General Assembly aim to enhance collective capacity and accountability, promoting an inclusive and evidence-based approach to preparedness and response (3). The WHO launched the Universal Health and Preparedness Review to drive accountability through regular intergovernmental dialogue between countries for health emergency preparedness (3,4). To ensure sustainable financing for HEPR, the WHO has launched the Pandemic Fund and is engaged in ongoing discussions within the G20 joint health and finance track. These efforts seek to transform financing mechanisms and establish surge financing for pandemic response (3). The WHO prioritizes the integrative and collaborative Public Health Surveillance Systems (PHSS) to be a practical tool to interconnect the national and global health systems that can strengthen the world’s preparedness to respond to global health emergencies in a timely manner. Recently, the WHO has released a report titled ‘Defining collaborative surveillance’ (5), which is the first component in a series aimed at strengthening HEPR (6). The report outlines specific objectives centred around reinforcing national surveillance systems for diseases, threats and vulnerabilities, improving laboratory and diagnostic capacities for pathogen and genomic surveillance and establishing collaborative capacities to predict, identify and assess risks while monitoring response efforts (5). The COVID-19 pandemic has exposed not only the weaknesses in the architecture of global health governance, but also the weaknesses in PHSS in nearly all countries, especially in low- and middle-income countries (LMICs) (7). Furthermore, the pandemic shed light on weaknesses in technical aspects and emphasized the critical need to prioritize the improvement of accountability and transparency. These weaknesses also include resource utilization, workforce efficiency, effective training and capacity building, infrastructural components such as technology and innovation and a robust information system needed for equitable sharing of public health surveillance data. Therefore, it is crucial to expedite the adoption of emerging tools and methodologies to enhance the accessibility of high-quality and up-to-date surveillance data (8) and promote equitable and inclusive distribution of resources to mitigate the impact of infectious diseases (9). The fragmentation and poor integration of PHSS on the local level and between countries and regions are evident and have negative effects on the mandate of the WHO. In this reflective theoretical analysis, we critically analyse the leadership of the WHO in the context of global health governance and the functioning of global health surveillance systems. The analysis explores this issue from two perspectives: policy shaping and effective implementation. Various aspects related to PHSS were examined, including the lack of consolidation and its impact on the WHO’s mandate in global health leadership. The rationale behind the need to address the PHSS issues is underpinned by the fact that it is a current global health priority and at the heart of all stakeholders’ health agendas, particularly in the aftermath of the COVID-19 crisis and climate change effects. All stakeholders, including the WHO, believe that global, regional and national PHSS is a critical element of the global health structure because it is a key component to identify global health threats, generate valid epidemiological data and take collective proper response. This perspective is undertaken to investigate the role and leadership of the WHO towards strengthening and consolidating the PHSS within the broader framework of global health governance. We aim to build a deeper understanding of complex issues of fragmentation and interconnectivity concerning both PHSS and the WHO governance.

## Method

This study employs a grounded approach, integrating a thorough examination of existing literature with the synthesis of expert perspectives. The review process encompassed a systematic thematic synthesis of 22 WHO publications and 21 peer-reviewed articles. The search strategy incorporated various sources, including Google Scholar for pertinent journals and studies, official state websites and portals for the latest reports and publications, and targeted searches for relevant grey literature. The review methodology was organized in three distinct phases: (1) initial exploration and selection of literature and studies based on precise criteria, prioritizing resources that were directly relevant to the topic and published since 2000; (2) rigorous data extraction focusing on key components of the subject and (3) comprehensive synthesis and thematic analysis of the extracted data to present the findings cohesively.

The thematic synthesis process involved a purposive assessment of these selected publications, employing critical appraisal techniques. This method ensured the presentation of pertinent findings supported by the authors’ insights, in line with the overarching objective of the review on global health governance and PHSSs. The synthesis process was executed using MS Word and Excel programs, guaranteeing accuracy and consistency across all stages. Additionally, expert perspectives were integrated into the analysis to provide a well-rounded and nuanced understanding of the subject matter.

## The landscape of global health governance

The global health governance was defined as ‘the use of institutions, rules, and processes to deal with challenges to health that require cross-border collective action to be addressed effectively’ (10). Effective governance plays a crucial role in attaining global health objectives. Recognizing this, the United Nations established the WHO with the purpose of providing global health governance as a strategic priority to seek the enhancement of constructive collaboration and adopt a more coordinated multisectoral approach to achieve a well-defined global health agenda (11,12). While the WHO has made commendable strides in advancing global health in various aspects, it has fallen short of meeting the expectations placed upon it in terms of its leadership role (12) and is insufficient in adequately addressing global health challenges (13). The fragmentation of the global health governance structure is emphasized, and this fragmented landscape is clear in three global domains: first, the global response to the COVID-19 pandemic (14), second, the attainment of the Sustainable Development Goals (SDGs), especially the health-related goals (15), and, third, the progress towards achieving Universal Health Coverage (UHC) (16). There is broad variance among the countries, particularly between high-income countries and LMICs, in response to the pandemic and in the level of progress towards the SDGs and UHC.

Furthermore, the global health governance encounters several challenges that are interconnected and often overlapping that necessitate a comprehensive and systemic approach to address them effectively. These challenges encompass the following: (i) insufficient efforts in harnessing and employing creativity, energy and resources; (ii) insufficient funding and lack of clear priority setting.; (iii) lack of robust accountability mechanisms and absence of transparency, monitoring and enforcement processes; (iv) limited coordination and collaboration among multiple stakeholders; (v) inattention to the fundamentals of health systems strengthening (12,16,17).

Addressing these challenges requires a holistic approach that considers their interdependencies and develops comprehensive strategies to tackle them collectively. By doing so, it is possible to enhance global health governance and make significant strides in improving health outcomes worldwide. One example of a holistic approach is the Global Action Plan for Healthy Lives and Well-being for All (SDG GAP), an initiative that was developed by the WHO and major global health organizations to enhance collaboration and collective actions in health, development and humanitarian response (15,18). In the recent progress report released in May 2023, the SDG GAP highlighted six recommendations: improve collaboration, maintaining GAP as a collaborative platform, enhancing collaboration at the country level in primary healthcare and climate resilience, implementing innovative approaches for impactful delivery, engaging with civil society, and strengthening incentives for collaboration through political leadership, governance direction and funding (18).

## Unveiling and enhancing the understanding of public health surveillance

Public health surveillance forms the epidemiological cornerstone for contemporary public health endeavours. The WHO defines it as ‘the continuous, systemic collection, analysis and interpretation of health-related data for use in public health actions to reduce morbidity and mortality and improve health’ (19). Surveillance systems provide valuable information for monitoring disease burden, detecting changes in occurrence or outbreaks, identifying risk factors and vulnerable populations, guiding public health actions, informing disease prevention and control programs and evaluating their effectiveness. The overarching goal of surveillance is to equip decision makers with timely and relevant evidence, enabling them to lead and manage more effectively (20,21).

Surveillance can be categorized into different types based on the format and content of data collection (22). Types of surveillance by forms of data collection: (i) passive or active surveillance: passive surveillance relies on voluntary reporting of data, while active surveillance involves proactive collection. Active surveillance typically generates high-quality data but requires substantial resources. (ii) Compulsory or voluntary: surveillance systems can be based on mandatory or voluntary data submission. (iii) Comprehensive or sentinel: comprehensive surveillance includes reports from the entire population within a specific geographical area. However, sentinel surveillance relies on notifications from selected healthcare facilities or institutions (22).

Types of surveillance by content of data collection: (i) indicator-based surveillance: this involves routine reporting of disease cases, including notifiable disease surveillance, sentinel surveillance and laboratory surveillance. Standardized information is collected to establish historical trends and trigger public health responses based on predefined threshold levels. (ii) Event-based surveillance: event-based surveillance focuses on the rapid detection, reporting, confirmation and assessment of public health events, including community-based surveillance, which is an active process that utilizes community representatives and health workers as contact persons for surveillance purposes. Both indicator-based and event-based surveillance systems are essential components of a national surveillance system and should complement each other (22,23).

Numerous global initiatives, frameworks and tools are playing pivotal roles in enhancing public health surveillance, early detection, and response to health threats worldwide. These include the Global Influenza Surveillance and Response System (GISRS) for collaborative virus data sharing (24), the Global Public Health Intelligence Network (GPHIN) for rapid threat assessment (25), and the Global Antimicrobial Resistance and Use Surveillance System (GLASS) for standardized antimicrobial resistance (AMR) tracking (26). Other key contributions include the Global Early Warning System for Major Animal Diseases (GLEWS+) (27), the Global Outbreak Alert and Response Network (GOARN) (28) and PulseNet International, dedicated to tracking foodborne infections (29). Furthermore, programmes such as ProMED-mail and the Asia Pacific strategy for emerging diseases (APSED III) contribute to infectious disease reporting and regional health security (30,31). These initiatives collectively facilitate interconnectedness, timely information sharing and coordinated action to address global health challenges.

## PHSSs in the context of global health governance

Numerous compelling pieces of evidence established a direct connection between the PHSS and the global health governance. A prominent illustration of this link is found in the International Health Regulations (IHR). Under the IHR, countries are required to establish and maintain surveillance systems capable of rapidly detecting and assessing public health events. These systems serve as early warning mechanisms for potential international health emergencies. By monitoring and reporting on disease outbreaks, surveillance systems contribute vital information to global health governance efforts, enabling coordinated responses and resource allocation at both regional and global levels (1,32). An additional line of evidence lies in the Global Health Emergency Preparedness and Response that describes the WHO’s role in global health emergency preparedness and response, particularly during pandemics and other public health emergencies. Public health surveillance is instrumental in guiding and informing the WHO’s actions, including risk assessment, decision-making and resource allocation, which are all essential elements of global health governance (3,33).

The PHSS is a fundamental pillar of the global health architecture. These systems are considered essential for the health information and scientific databases that can lead to effective and efficient global public health decision-making and appropriate public health actions (7,34). It is evident that these surveillance systems need to be consolidated, integrated and institutionalized into the national health systems as they are central to the successful achievement of the SDGs, UHC and pandemics responsiveness (35). Therefore, the WHO is placing greater emphasis on prioritizing PHSS as a valuable tool of global health policy and actions, and the strategic direction of the WHO is to ensure that global, regional and national surveillance systems are effectively functioning and integrated. Although evidence indicates that PHSSs are present in numerous countries, these systems often exhibit a fragmented structure as they are primarily designed for disease-specific programmes, reliant on international donor funding, and face challenges related to financial and human resources sustainability, as well as fluctuation (33,34). This specific fragmentation and disintegration in the PHSS are mainly reported in the LMICs and regions such as Africa, the Middle East and Asia (34). The COVID-19 pandemic has exposed and amplified this significant gap and weaknesses in the global health landscape where the existing surveillance systems impeded the early identification and response to COVID-19 cases and hindered any effective and rapid containment strategies (7). Furthermore, the lack of organization and structural framework hampers the collection, sharing, reporting and analysis of epidemiological data within the surveillance systems. Many existing disease surveillance systems are inadequate in effectively measuring the health impact of outbreaks and are insufficiently designed, resourced and operated to detect outbreaks for timely and effective public health interventions (34).

The Technical Framework in Support of IHR (2005) Monitoring and Evaluation: Joint External Evaluation Tool (36) provides criteria for effective surveillance as part of the broader assessment of countries’ capacities to implement the IHR with targets to (i) strengthen surveillance systems to detect significant events for public health and health security effectively, (ii) foster communication and collaboration among sectors, national and international authorities, and (iii) enhance national and regional capacity to analyse data incorporating the use of interoperable and interconnected electronic tools. Nevertheless, the implementation of these criteria may be impeded by the challenges associated with global health governance fragmentation. These challenges have been highlighted by different global health organizations and there is a pressing need to tackle them. This would support the countries in establishing strategies for integrated disease surveillance and enable them to overcome data fragmentation in any context (34). These challenges are:

1. *Lack of leadership and governance*. Strong leadership is crucial as the primary and indispensable prerequisite for realizing shared visions and advancing progress towards a more cohesive and resilient global PHSS (34).
2. *Lack of priority and imbalance in coverage of surveillance systems*. A vision for global health surveillance to address the most critical health problems and diseases with a focus on low-resource settings is essential. These systems are particularly important for population health and major diseases such as the non-communicable diseases (NCDs) (37). For example, NCDs are heavily affecting the health and wealth of the population with high prevalence and economic burden. NCDs and their associated risk factors, including socio-economic and environmental risk factors, should be integrated in the structure of the PHSSs (38). This priority-driven vision can tackle the fragmentation of surveillance between disease-specific programmes’ that are rarely grouped under an integrated and comprehensive national health information system (34).
3. *Inadequate innovative technology and digital solutions and fragmented data systems*. To a considerable extent, this challenge has been observed in LMICs, as most of these countries still rely on paper-based broken systems. Additionally, the disease-specific programmes’ surveillance systems usually use different information technology applications, which limits the opportunity to combine them under one comprehensive national surveillance system. However, in settings where electronic reporting systems are used, these systems are non-interoperable, and therefore cannot exchange data with other systems (34).
4. *The lack of information resources, robust surveillance framework for implementation, and limited and unsustainable financial resources* (34,39). These frameworks are deficient in LMICs with scarcity of financial resources. However, these countries benefited from disease-specific surveillance programmes with specific funding sources that leave gaps in the national surveillance opportunities, duplicate efforts and waste resources. This also may apply to the challenges of availability and competency of human resources required to operate the PHSSs in a collaborative and effective manner.

## Fragmentation and interconnectivity of global health governance and PHSSs

The impact of complex and fragmented global health governance on the organization and integration of PHSSs at both the global and national levels is evident. This situation significantly hinders the WHO capacity to effectively lead, guide and support a robust global health governance system. The primary responsibility of the WHO is to establish cohesive global and national PHSSs as operational tools to enhance global, regional and national capabilities in preventing and controlling infectious diseases. However, the current fragmented global systems undermine the effectiveness, integration and collaboration of most existing surveillance systems, rendering them inadequate and non-functional. The fragmentation of surveillance systems inhibits the information and data from being routinely and systematically shared with the WHO to then take data-driven and evidence-based decisions. Moreover, this may make the WHO’s mission difficult to lead and effectively guide the global health actions of combating disease outbreaks, epidemics and health issues. Evidence revealed that the response to the Ebola epidemic was inadequate, and the Director-General of the WHO underlined the importance of regaining the trust of the global community in the WHO’s ability to manage global health crises and disease outbreaks (40). There is an obvious indication that the WHO still needs to take additional actions for effective global health leadership, including supporting the Member States in building and strengthening consolidated, collaborative and integrated PHSSs.

However, to date, there is some progress made in the area of the IHR implementation, for example, the introduction of national focal points to connect with different government sectors, stakeholders and the WHO; an increase in reporting transparency; improvement in the use of early warning systems; and enhancement of cooperation between organizations dealing with human and animal health (41). A notable example of this progress is the establishment of the WHO Hub for Pandemic and Epidemic Intelligence, which works closely with Member States and WHO Regional and Country Offices to strengthen their data-sharing capacities and enable partners from around the world to collaborate and co-create tools to gather and analyse data for early warning surveillance (42). Nevertheless, there are still significant gaps that necessitate a revision of the IHR with a focus on surveillance systems integration.

Case Studies 1 and 2 play a pivotal role in substantiating the theoretical underpinnings of this analysis. Case Study 1, which examines the Ebola outbreak in West Africa, vividly illustrates the repercussions of ineffective implementation of health surveillance systems within a fragmented governance structure. This case underscores the critical importance of strategic planning, coordination and robust surveillance infrastructure in effectively responding to public health crises. Case Study 2, focused on Rwanda’s Integrated Disease Surveillance and Response programme, stands as a testament to the transformative potential of cohesive public health surveillance systems guided by sound governance principles. Rwanda’s success story highlights the substantial positive impact that concerted efforts, investment in health information infrastructure and strong inter-sectoral collaboration can have on strengthening national surveillance capacities. Together, these case studies provide empirical grounding for the argument that a consolidated and well-integrated PHSS, underpinned by effective global health governance, is indispensable in effectively addressing emerging health threats on a global scale.

**Case Study 1: Fragmentation of global health governance and PHSS**

The case of the Ebola outbreak in West Africa serves as an example where ineffective implementation of a health surveillance system can be attributed to governance failure (43), particularly in Guinea, Sierra Leone and Liberia between 2014 and 2016. That was noticed through: (i) The governments of the West African countries were lacking preparedness and planning to handle the Ebola outbreak. There was a lack of strategic planning and coordination between government agencies, resulting in delays in response and containment efforts. (ii) Weak health infrastructure and lack of investment and poor governance in the health sector. (iii) Inadequate surveillance systems with scarce necessary resources and technology for timely detection and reporting of cases. (iii) Corruption and lack of accountability along with mismanagement and misallocation of resources for the Ebola response. (iv) Insufficient coordination and non-effective collaboration between international organizations, governments and non-governmental organizations, which hampered the implementation of a unified surveillance system.

**Case Study 2: Interconnectivity of global health governance and PHSS**

Rwanda is a successful example of implementing a PHSS as part of a strategy for good governance through the ‘Integrated Disease Surveillance and Response’ (IDSR) programme (44,45). Rwanda has made noteworthy progress in improving its public health infrastructure and surveillance systems. The IDSR programme was initiated as part of the government’s commitment to good governance and effective public health management. The key elements of its success are: (i) the Rwandan government developed a *comprehensive strategy and policy framework* to guide the implementation of the IDSR programme. (ii) The government invested in *strengthening health information systems* by establishing a centralized electronic reporting system. (iii) The success of the IDSR programme was attributed to *strong collaboration and coordination* between different sectors. The government worked closely with the Ministry of Health, other relevant ministries, international partners and local communities to ensure the effective implementation of surveillance activities and timely response to public health threats. (iv) *Continuous monitoring and evaluation* were integral to the programme’s success. The government established a system for monitoring surveillance activities, data quality and the overall performance of the programme. Feedback mechanisms were implemented to address challenges and improve the system over time (44,45).

The focus of this perspective centred around four major problems that are limiting the WHO’s ability to perform its duties. The problems are linked to the malfunctioning of global health surveillance systems. (i) Lack of integrated PHSS, particularly in LMICs, which inhibits the data-driven decision-making process to tackle outbreaks and diseases. (ii) The structural and operational fragmentations and the cost associated with addressing them, which will expose the WHO to financial stress and weaken the prospect for greater autonomy (46). (iii) Conflicted political, technical and economic interests and priorities among Member States, agencies and networks (40). Lastly, (iv) lack of longstanding and sustainable One Health collaborations, which increases the burden on the WHO’s ability to provide policy guidance, technical support and resource mobilization (47).

Nevertheless, the WHO believes that collaboration is an essential approach needed for robust surveillance systems. One example is the collaboration that produced the One Health Approach with the Food and Agriculture Organization of the United Nations, the World Organisation for Animal Health, and the United Nations Environment Programme. This new operational approach was articulated to address the global health issue of AMR through establishment of multisectoral coordination mechanisms, the recent launch of global research agenda for AMR (48) and the formation of surveillance and information sharing systems (49).

## Conclusion

The global health governance landscape continues to be weak, fragmented and unstructured due to the following factors: the multiplicity of global health actors, drawbacks in global leadership, divergent interests and priorities, lack of accountability, and power dynamics. It is also evident that there is fragmentation in public health surveillance at global, regional and local levels and this is associated with key challenges such as lack of leadership, lack of priorities alignment, imbalance in coverage of surveillance systems, inadequate innovative technology and digital solutions, and fragmented data systems.

Overall, the fragmentation of global health governance and of PHSSs represents two sides of the same coin. They require equal attention as they are negatively affecting the WHO’s ability in decision-making processes, funding sustainability, resource allocation, global health priorities alignment at all levels and in the implementation of a One Health collaboration approach.
